# Geographical distribution of *Gyrodactylus salaris* Malmberg, 1957 (Monogenea, Gyrodactylidae)

**DOI:** 10.1186/s13071-020-04504-5

**Published:** 2021-01-09

**Authors:** Giuseppe Paladini, Andrew P. Shinn, Nicholas G. H. Taylor, James E. Bron, Haakon Hansen

**Affiliations:** 1 Institute of Aquaculture, Faculty of Natural Sciences, University of Stirling, Stirling, FK9 4LA Scotland, UK; 2 Benchmark R&D (Thailand), No. 57/1 Moo 6, Samed Sub-District, Muang Chonburi District, Chonburi, Chonburi Province 20000 Thailand; 3 CEFAS Weymouth Laboratory, Barrack Road, Weymouth, DT4 8UB UK; 4 Norwegian Veterinary Institute, PO Box 750 Sentrum, Oslo, N-0106 Norway

**Keywords:** Monogenean, OIE, Salmonidae, Pathogen, Spread, *Salmo salar*, Parasite

## Abstract

**Background:**

*Gyrodactylus salaris* Malmberg, 1957 is an OIE (Office International des Epizooties)-listed parasitic pathogen and had until the current study been reported from 19 countries across Europe, although many of these records require confirmation. The last comprehensive evaluation regarding the distribution of *G. salaris*, however, was made in 2007, although some of the states identified as being *G. salaris*-positive were ascribed this status based on misidentifications, on partial data resulting from either morphological or molecular tests, or from records that have not been revisited since their early reporting. It is thus important to go through the reports on *G. salaris* to obtain a status for each country.

**Methods:**

To provide a revised update of the *G. salaris* distribution, a literature review was necessary. This literature, however, was not always readily accessible and, in certain cases, the article only made superficial reference to the parasite without providing details or data to support the identification. In most cases, the original specimens were not deposited in a national collection. Additional *Gyrodactylus* material for the current study was obtained from selected salmonid populations with the aim to contribute to current understanding regarding the distribution of *G. salaris*. Additional parasite material collected for this study was processed following standard procedures for species identification in *Gyrodactylus* [1].

**Results:**

From the work conducted in the current study, *G. salaris* is reported from a further three regions in Italy, alongside three other species, and appears to occur extensively throughout central Italy without causing significant mortalities to its rainbow trout, *Oncorhynchus mykiss* (Walbaum), host. The analysis of archive material from *G. salaris*-positive farms would suggest that *G. salaris* has been in this country since at least 2000. Material obtained from rainbow trout from Finland and Germany are confirmed as *G. salaris*, supporting existing data for these countries. No specimens of *G. salaris*, however, were found in the additional *Gyrodactylus* material obtained from rainbow trout reared in Portugal and Spain. A morphologically similar species, *Gyrodactylus teuchis* Lautraite, Blanc, Thiery, Daniel et Vigneulle, 1999, however, was found.

**Conclusions:**

Following the present review, *Gyrodactylus salaris* is reported from 23 out of 50 recognised states throughout Europe; only records from 14 of these states have been confirmed by either morphology and/or by an appropriate molecular test and are considered valid, while only nine of these records have been confirmed by a combination of both methods. 
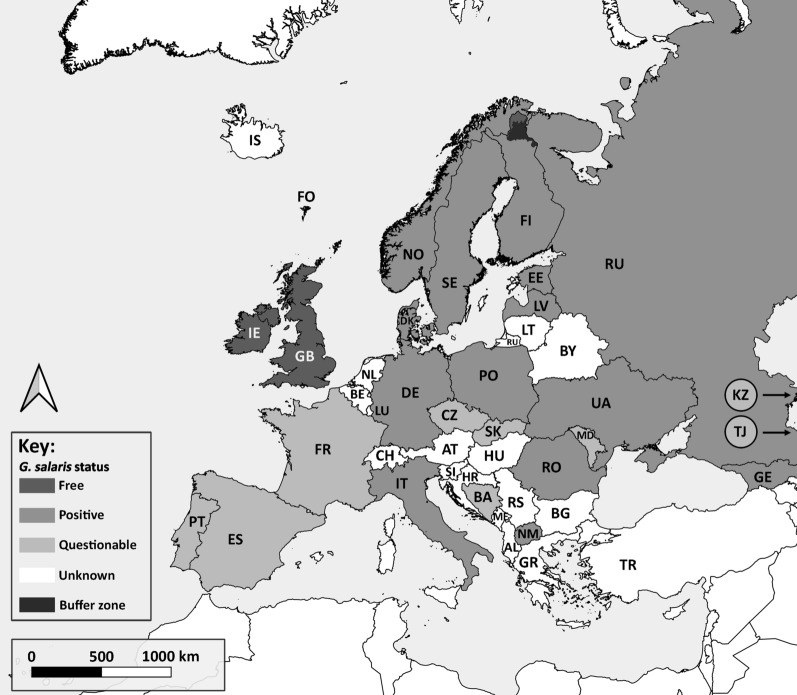

## Background

*Gyrodactylus salaris* Malmberg, 1957 (Monogenea) is endemic to the areas east of the Baltic sea [2] and has been shown to be pathogenic to the Atlantic strain of Atlantic salmon, *Salmo salar* L., and to a lesser degree to the Baltic strain [3–5]. *Gyrodactylus salaris* has been reported from at least eight salmonid hosts (Table 1), and according to Chapter 2.3.3 of the Aquatic Animal Health Code (Aquatic Code) published by OIE (Office International des Epizooties) [6], the species that fulfil the criteria for listing as susceptible to infection with *G. salaris* include: Arctic char, *Salvelinus alpinus* (L.), Atlantic salmon, *S. salar*, brown trout, *Salmo trutta* L., grayling, *Thymallus thymallus* (L.), North American brook trout, *Salvelinus fontinalis* (Mitchill, 1814), and rainbow trout, *Oncorhynchus mykiss* (Walbaum). The occurrence of *G. salaris* on rainbow trout, a species that is traded extensively across Europe, is of particular concern [7, 8]. Rainbow trout have been demonstrated to be susceptible to *G. salaris* infection, and although these infections in some instances can be self-limiting, they can persist for up to 90 days or more [9]. Low levels of infection and the absence of evident clinical signs could mean that infections go undetected in a consignment of fish [7, 8]. This, coupled with the ability of hosts to carry an infection for long periods, increases the window of exposure and raises concerns regarding the movement of rainbow trout, in terms of their potential role as a carrier and source of *G. salaris* infection of other susceptible fish populations, across Europe.

**Table 1:** List of countries from which *Gyrodactylus salaris* has been reported to occur on salmonids

Country	Host^a^	ID status	Year of first confirmed detection	Method of ID^b^	Presence in GenBank	Representative references
Bosnia-Herzegovina (BA)	*Om*,* Ss*,* St*,* So*	Unconfirmed	–	A	No	[112, 114, 150]
Czech Republic (CZ)	*St*	Unconfirmed	–	A + B	No	[34, 61]
Denmark (DK)	*Om*,* Ss*	Valid	1997	A + B	Yes	[96^d^, 99, 100]
Estonia (EE)	*Ss*	Valid	2010	B	Yes	[106]^d^
Finland (FI)	*Om*,* Ss*	Valid	1984	A + B	Yes	[88, 91^d^, current study]
France^c^ (FR)	*Om*	Unconfirmed	–	A	No	[127]
Georgia (GE)	*St*	Valid	1978	A	No	[31, 39^d^]
Germany (DE)	*Om*	Valid	1990	A + B	No	[24, 94^d^, 95]
Italy^c^ (IT)	*Om*	Valid	2000	A + B	Yes	[32^d^, current study]
Kazakhstan (KZ) or Tajikistan (TJ)	*Sto*	Unconfirmed	–	A	No	[129]
Latvia (LV)	*Ss*	Valid	2002	B	Yes	[13^d^]
Moldova (MD) (including Transnistria)	*St*	Unconfirmed	–	A	No	[31]
North Macedonia (NM)	*Oml, Sl*	Valid	2007	B	Yes	[88^d^, 104]
Norway (NO)	*Ss*,* Sa*,* Om*	Valid	1975	A + B	Yes	[13, 26, 68^d^, 138, 151, 152]
Poland (PO)	*Om*,* St*	Valid	2006	A + B	Yes	[102^d^]
Portugal^c^ (PT)	*Om*	Unconfirmed	–	A	No	[127]
Romania (RO)	*Om*,* St*,* Sf*	Valid	2008	A + B	Yes	[15^d^]
Russia (RU)	*Ss*	Valid	1972	A + B	Yes	[16, 39^d^, 88]
Slovakia (SK)	*St*	Not valid	–	A	No	[37, 39]
Spain^c^ (ES)	*Om*	Unconfirmed	–	A	No	[125]
Sweden (SE)	*Ss*	Valid	1951	A + B	Yes	[13, 16, 26, 42^d^]
Ukraine (UA)	*Om*,* St*	Valid	1960	A	No	[59, 61]
United Kingdom (UK) (including Northern Ireland)	*St*	Not valid	–	A	No	[109^d^]

Some of the records are questionable, and the confirmation of the presence of *G. salaris* needs further verification. For each country, only the mainland is considered; larger island groups are considered separately

^a^*Om*, *Oncorhynchus mykiss*; *Sl *, *Salmo letnica*; *So *,* Salmo obtusirostris*; *Ss*, *Salmo salar*; *Sto*, *Salmo trutta oxianus*; *St,Salmo trutta*; *Sf*,* Salvelinus fontinalis*;* Sa*,* Salvelinus alpinus*

^b^Method of identification: A, morphology only; B, molecular only; A + B, morphology + molecular characterisation.

^c^Large islands, such as Corsica (France), Sardinia and Sicily (Italy), Balearic and Canary Islands (Spain) or Madeira (Portugal) are included, although their *G. salaris* status should be considered separately from their respective mainland territories

^d^Date of first publication

In the current *Manual of Diagnostic Tests for Aquatic Animals*, the OIE states that the preferred method for the diagnosis of *G. salaris* is molecular analysis but that morphological analysis alone is also accepted [6]. The molecular analyses consist of sequencing of the ribosomal internal transcribed spacer region (ITS) and the mitochondrial (mt) cytochrome* c* oxidase subunit I (COI) gene. Sequencing of the ITS alone establishes the species status (but see the discussion on *G. salaris vs* *G. thymalli* later in this article), while sequencing and phylogenetic analysis of COI is applied to assign a sequence to its nearest known relative. Reports of *G. salaris* from before the implementation of molecular methods were of course based solely on data derived from morphological investigations, but can nevertheless be considered valid if the reports contain images or drawings that are of a sufficiently high quality (most notably images of the marginal hook sickles) for re-evaluation. New findings should also be confirmed by the OIE reference laboratory, but very few findings have been submitted to OIE for confirmatory analyses (with Sweden and lately Russia as noticeable exceptions). Ideally, whole individuals from a new finding/locality should be submitted to the OIE reference laboratory as EtOH-preserved specimens. DNA extracts of the original new finding and/or morphological preparations can also be submitted for analyses and evaluation by the reference laboratory.

The difficulty in discriminating *G. salaris* from the benign *G. thymalli* Žitňan, 1960 has been stressed and debated over by many authors [10–18]. When morphometrics alone is considered, it was suggested earlier that subtle differences in the marginal hook sickles could permit the discrimination of these two species [11, 19, 20]. These studies were, however, based on a limited number of samples, and host information is required to support identification. Olstad et al. [17], looking at a data set of 168 specimens collected from ten populations infecting four different hosts and analysing the morphological characteristics of the opisthaptoral hard parts of the specimens (removing the effect of size), suggested that an* a priori* species delineation based on host alone is not possible and that more information is required to support identification. The study of Shinn et al. [21] also demonstrates that misclassifications can occur when all supporting information is removed and morphology experts are asked to make an identity based on the specimen only (*i.e.* no data relating to host or location). Although the ITS region of the rRNA gene is frequently used to describe and discriminate most *Gyrodactylus* species, this region is almost invariable between *G. salaris* and *G. thymalli* and thus cannot be used to distinguish between these two species [22, 23]. Other genomic regions that have been used to facilitate the discrimination between these two species include sequences of the intergenic spacer (IGS) and COI [12–16, 24–26]. Analyses of COI sequences have revealed the presence of a high number of haplotypes (see [14 and 15] for a summary) which generally allow for grouping according to host specificity and/or geography (drainages). Thus, although both COI and IGS sequences show more variation than the ITS, they do not support a separate species status for *G. salaris* and *G. thymalli*. In addition, Fromm et al. [18] analysed microRNA from a limited number of populations of *G. salaris*/*G. thymalli* and suggested, based on the results, that the two species are conspecific.

Thus, the most recent studies based on analyses of molecular data, both mtDNA [13, 14, 16, 27] and microRNA [18], support synonymising the two species, and recently all records of *G. thymalli* in the database of The National Center for Biotechnology Information (https://www.ncbi.nlm.nih.gov/) have been synonymised (*i.e.* all records are now listed as *G. salaris*).

Despite the morphological and genetic similarities, *G. thymalli* appears to be restricted to grayling, whilst *G. salaris* has never been recorded from grayling in nature [28], although this again depends on how the species status is assigned. In rivers where both Atlantic salmon and grayling are present, however, the parasite strains (haplotypes) on each host do not seem to overlap [29, 30]. In addition, the parasites infecting grayling are assumed to be non-pathogenic to Atlantic salmon, and thus records from grayling are not included in this report. It is also worth mentioning that specimens from grayling, if these should not be previously known COI variants (haplotypes) identified as *G. salaris*, are still not reported as *G. salaris* by the OIE. Thus, in effect, the OIE manual follows a host-based diagnosis (in short, where parasites from grayling are named *G. thymalli* and those from other hosts are named *G. salaris*) until new markers that can differ between pathogenic and non-pathogenic strains are available.

Although in the current study we comment on whether the *G. salaris*-positive status of each country is valid, we do not debate the validity of other species of *Gyrodactylus*-parasitising salmonids. We therefore do consider the distribution of *G. thymalli* (*s.s.*) from grayling throughout Europe, although from previous studies it would appear to be widespread [13, 14, 16, 26]. For the present study, *G. salaris* is defined as specimens morphologically and molecularly diagnosed as *G. salaris* and not infecting grayling, and *G. thymalli* is defined as specimens parasitising grayling only.

The aim of this study is to provide a revised update of the distribution of *G. salaris* for each European state, supplemented and supported by the analysis of additional *Gyrodactylus* specimens collected from some salmonid populations from certain European states with an unconfirmed status for *G. salaris* infection. Although the European distribution of *G. salaris* has been discussed several times in the scientific literature [2, 15, 31, 32], an updated revision is necessary. This is because the status of certain countries has been reported as being *G. salaris* positive but on re-examination appears to have been based on misidentifications of morphologically similar species, whilst the presence of *G. salaris* in other countries have been reported since the last major review [2]. To help understand the existing distribution and records of *G. salaris* across Europe, Table 1 presents codes for each *G. salaris*-positive country by year of first detection and the diagnostic method used to characterise the record is presented .

## Methods

To provide a revised update of *G. salaris* distribution across Europe, we undertook a literature review using information from a range of white and grey literature, including peer-review academic journals and institutional reports. This literature, however, was not always readily accessible and, in certain cases, the article only made superficial reference to the parasite without providing details or data to support the identification. In most cases, the original specimens were not deposited in a national collection. The historical records for each country where *G. salaris* has been reported, officially or unofficially, are listed in "Results" section, in chronological order for the year it was first published, rather than diagnosed, for that particular country and these are then summarised in Table 1.

To further investigate the *status inquirendae* for the presence of *G. salaris* in certain European states and to supplement current understanding, additional salmonid samples were collected and screened. The results from each of these additional samples will be commented upon under the entry for each country. The new parasite material considered here, and which is not reported elsewhere, includes specimens from rainbow trout from Italy, Portugal and Spain collected between 2008 and 2010.

The current criteria used to diagnose *G. salaris* today follow those detailed in the OIE manual [1], where diagnosis can be a two-step approach using a combination of morphological evaluation of the attachment hooks and molecular methods. The preferred method for the diagnosis of *G. salaris*, however, is molecular analysis. In assessing the validity of each *G. salaris* country report, we applied the following criteria: (i) whether OIE guidelines have been followed; (ii) whether the morphological investigations were conducted by recognized *Gyrodactylus* experts and the associated report and images of the attachment hooks are of sufficient quality to permit a robust, independent assessment; (iii) whether additional information (such as the intensity of infection or host details) lend support to the report of *G. salaris*.

### Morphological analysis

The specimens collected were prepared for both morphological and molecular analyses following the methods detailed in Paladini et al. [32] and Shinn et al. [20, 21]. When unmounted parasites were available, gyrodactylids were cleaned of extraneous mucus using mounted triangular surgical needles (size 16; Barber of Sheffield, Sheffield, UK) and observed under an Olympus SZ40 dissecting microscope (Olympus Corp., Tokyo, Japan) at ×4 magnification. Each individual specimen was then transferred to a glass slide and cut in half with a scalpel blade. The anterior part was transferred to a 1.5 ml Eppendorf containing 95% ethanol for subsequent molecular characterisation. The posterior part of the specimen, containing the attachment organ, was subjected to proteolytic digestion to remove tissue surrounding the attachment hooks, following the method detailed in Paladini et al. [32] which is a modification of the protocol given in Harris and Cable [33]. Tissue digestion was arrested and sclerites mounted in situ by the addition of 2 µl of a 1:1 saturated ammonium picrate:100% glycerine mix solution. The edges of the coverslip were then sealed with common nail varnish to make a semi-permanent mount. The digested specimens were then photographed using a JVC KY-F30B 3CCD camera with an interfacing ×2.5 top lens fitted to an Olympus BH2 compound microscope with phase contrast (Olympus Corp.).

### Molecular analysis

The corresponding upper parts of the parasite bodies, previously stored in 95% ethanol, were subjected to molecular characterisation at the Norwegian Veterinary Institute (Oslo, Norway). DNA was extracted from specimens collected from Portugal using DNeasy® Blood & Tissue minikit (Qiagen, Hilden, Germany). To amplify (PCR) a fragment spanning the 3′ end of the 18S ribosomal RNA subunit, internal transcribed spacers 1 and 2 (ITS1 and ITS2), the 5.8S subunit and the 5′ end of the 28S subunit, the primer pairs ITS1A (5′-GTAACAAGGTTTCC GTAGGTG-3′) and ITS2 (5′-TCCTCCGCTTAGTGATA-3′) [34] were used. The PCR reactions were performed with PuReTaq Ready-To-Go™ PCR beads (GE Healthcare, Chicago, IL, USA) following the manufacturer’s instructions. The PCR program was as follows: 95 °C, 4 min; then 95 °C/min, 55 °C/min, 72 °C/2 min for 35 cycles. PCR products were then purified using a NucleoSpin® Purification kit (Macherey–Nagel GmbH & Co. KG, Düren, Germany), and sequencing reactions were carried out on a MegaBACE 1000 analysis system (GE Healthcare) using DYEnamic ET dye terminators. For sequencing, the internal primers ITS4.5 (5′-CATCGGTCTCTCGAACG-3′) [34], ITS1R (5′-ATTTGCGTTCGAGAGACCG-3′), ITS18R (5′-AAGACTACCAGTTCACT CCAA-3′), ITS2F (5′-TGGTGGATCACTCGGCTCA-3′) and ITS28F (5′-TAGCTCTAG TGGTTCTTCCT-3′) [35] were used in addition to the PCR primers. The obtained sequences (ITS1, 5.8S and ITS2 only) were proofread and assembled in Vector NTI 11 (Invitrogen, Carlsbad, CA, USA) and subjected to a BlastN search [36]. The additional *Gyrodactylus* material acquired from Finland and analysed by molecular methods is reported on in [21].

## Results

**Fig. 1:**
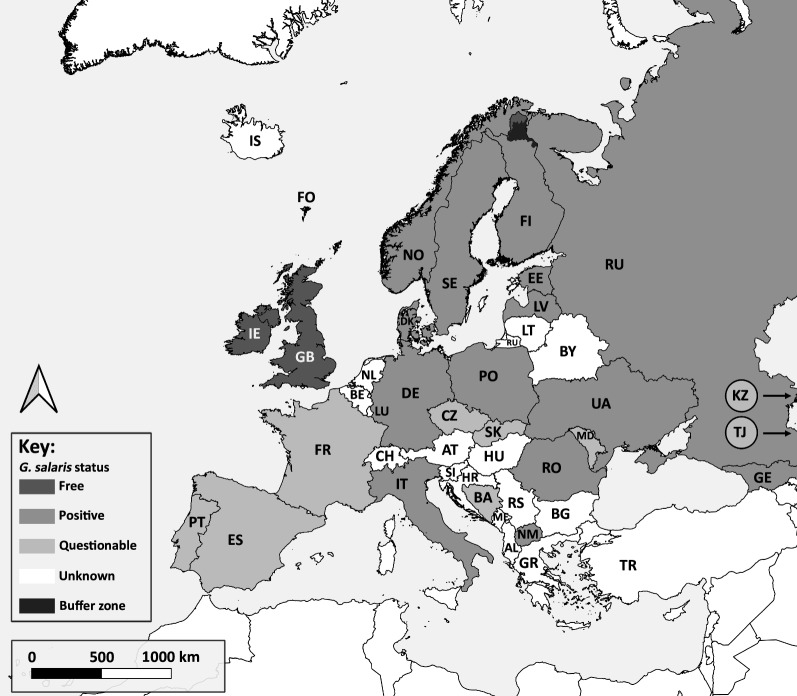
Map highlighting *Gyrodactylus salaris*-positive states (medium-grey colouration). For territories such as France, Italy, Spain and Portugal only the status of the mainland is considered and larger islands (e.g. Balearic, Canary, Corsica, Sardinia, Sicily, etc.) under their respective sovereignty are considered as separate geographic entities. For the purposes of this study, Kaliningrad, the Russian exclave, is considered as a separate geographic zone to the main Russian state. The reports of *G. salaris* from Kazakhstan (or Tajikistan), France, Lithuania, Portugal, Slovakia and Spain (light-grey colouration) are questionable and need further verifications regarding the presence or absence of *G. salaris*. The Republic of Ireland and the UK (including Northern Ireland) are the only two countries currently declared *G. salaris*-free, as are the water catchments of the Tenojoki and Näätämönjoki in Finland (dark-grey colouration). The catchments of the Paatsjoki, Luttojoki and Uutuanjoki are considered as buffer zones (black colouration). Countries where the status of *G. salaris* is unknown are left in white. Country codes are given in Table 1

A re-evaluation of the reports regarding the distribution of *G. salaris* across Europe in the present study showed that reports of the species are currently validated from 14 countries throughout Europe, although its presence has been reported from 23 countries (Fig. 1; Table 1). The records from nine countries are not considered valid or are unconfirmed. The report from Slovakia (see [37, 38]) is not considered valid (see [39]), whilst the identity of the specimens recovered from Bosnia-Herzegovina, Czech Republic, France, Moldova, Portugal, Spain and Transnistria and the record from either Kazakhstan or Tajikistan (see below) are questionable and their *G. salaris* status requires further re-examination. The parasite species reported from France, Portugal and Spain was most likely *Gyrodactylus teuchis* Lautraite, Blanc, Thiery, Daniel *et* Vigneulle, 1999 (see [40, 41]), a species bearing some morphological similarities to *G. salaris*, but undescribed at the time of the original “*G. salaris*” report for each of these countries. The apparent absence of *G. salaris* from France, Portugal and Spain is interesting given that it is found in Italy and Romania on *O. mykiss*. The basis for this absence is not known and whether this is due to robust border biosecurity screening and quarantine procedures that have prevented entry. Alternatively, infections may exist but at low levels that have not posed an impact to fish health and, therefore, not have been detected under routine health screening.

The *G. salaris* records for each European state are divided below into three sections (i.e. known *G. salaris*-positive European states; *G. salaris*-free states; and states where the *G. salaris* status requires confirmation) and are discussed chronologically by the date of their first official announcement. The acquisition of new *Gyrodactylus* material from Finland, Germany, Italy, Portugal and Spain, and its subsequent examination, is discussed under each corresponding European state. A summary table listing the validity of the *G. salaris* for each country is presented in Table 1. Figure 1 highlights the other European states for which no information on the status of *G. salaris* is available.

### Known *G. salaris*-positive European states

The countries below are listed by the year the first official report was published.

#### 1957–Sweden

In 1951, Professor Göran Malmberg at the University of Gothenburg received a sample of *Gyrodactylus* collected from Atlantic salmon held at the experimental fish farm station in Hölle (now Hölleforsens Laxodling) situated on the Indalsälven River, Sweden, draining into the Baltic Sea. The findings from this material were reported on 6 years later, when the taxonomic description of *G. salaris* was published, based on one single specimen [42]. In 1954, the salmon parr held at the Hölle farm were observed to harbour a heavy *Gyrodactylus* infection. This was the first observation regarding the differential sensitivity and susceptibility of two Atlantic salmon stocks, the Atlantic and Baltic strains, to *G. salaris* ([43], see also [2]). *Gyrodactylus salaris* is likely present in all Baltic salmon rivers ([43]; E. Degerman personal communication). The parasite was first found on the Swedish west coast in 1989 [44] and has since then been recorded from 16 rivers draining into the Kattegat and Skagerak [44–51]. The parasite has continued to spread on the Swedish west coast and both in 2015 and 2016 new infections were detected in the Rolfsån and Kungsbackaån rivers, Halland county. The Göta River and its tributaries represent the northernmost occurrence on the Swedish west coast and, based on the higher salinity (> 20 promille/ppm) in the sea north of this river, the risk of further natural spreading is considered quite low.

The parasites found in the Swedish rivers draining into the North Sea represent several mitochondrial haplotypes [13] and are suggested to originate from the Baltic Sea. *Gyrodactylus salaris* might have been introduced to the west coast by stocking of infected fish and has spread further by brackish water migration of the host [13, 52, 53]. The parasites in the Göta älv River and its main source, Lake Vänern, carry unique and divergent haplotypes [13] not found elsewhere that might have been infecting the landlocked salmon since its isolation when Lake Vänern was created after the Quaternary glaciation 10,000 years ago. The presence of these unique haplotypes indicates that the infection in the Göta älv River is not the source of infection to the other rivers along the Swedish west coast.

*Gyrodactylus salaris* is believed to occur naturally in Swedish river systems draining into the Baltic Sea and is therefore not considered to be pathogenic in the wild, as host mortalities associated with the presence of the parasite have never been reported [44–46] and *G. salaris* do not appear to be particularly pathogenic to hosts from these populations ([54–56], but see [5]). In rivers on the Swedish west coast, however, there are records of high intensities of infection. In 1998, Alenäs and colleagues [52, 57] reported a 90% decrease in the salmon parr density from the Säveån River, a tributary to the Göta älv River, which were infected with high burdens (approx. 1,700 specimens fish^−1^) of *G. salaris*. The mitochondrial haplotype A of *G. salaris*, which is widespread and pathogenic throughout Norway, has also been found on the Swedish west coast, such as in the Ätran and Surtan rivers [13], suggesting that this pathogenic haplotype is not confined geographically. Indeed, high intensities of infection with this parasite strain have been reported from the Ätran River [52, 58].

In conclusion, there are multiple reports of the occurrence of *G. salaris* from Sweden, many of which have been confirmed by both morphological and molecular analyses (Table 1). The record of *G. salaris* is valid.

#### 1967–Ukraine

A parasitological survey on 295 fish sampled from two Ukrainian rivers, the Tisa and the Seret, found *G. salaris* on brown trout, *S. trutta*, collected from the Seret River [59]. Later in 1973, Malmberg [60] reported finding *G. salaris* on *S. trutta* collected from a Carpathian hatchery, and although the exact location of the hatchery was not specified at the time, in a later account Malmberg [31] indicated that these represented specimens that had been donated by Dr. Kulakovskaja to Prof. Malmberg back in 1960, and originated from the Seret River. Further records of *G. salaris* result from an investigation conducted by Tesarcik and Ivasik [61] on brown trout and rainbow trout sampled from a number of Carpathian ponds. The authors reported finding *G. salaris* on both hosts from ponds fed by the Dniester and Danube rivers, within the Ukraine [61].

In 1983, Ergens [39] described *Gyrodactylus* sp. material collected from the fins of *S. trutta* from two localities within the Autonomous Republic of Crimea, Ukraine. The first sample was taken in 1975 from the Salgir River, whilst the second sample, collected in 1976, was from the Angara River [39]. Ergens remarked that the specimens most closely resembled *G. salaris* and *G. thymalli* but that a comparative analysis using a large number of specimens was needed to facilitate the identity of the specimens. Ten years later, Malmberg [31] suggested that *Gyrodactylus* sp.* sensu* Ergens, 1983 was a synonym of *G. salaris*. In a recent paper, Matvienko et al. [62] documented *G. salaris* as being present on *O. mykiss*, *S. fontinalis* and *S. trutta*, this identification, however, has since been rejected by the author as *G. birmani* Konovalov, 1967 (personal communication).

In conclusion, as there is agreement between Ergens indirectly and Malmberg, the report of *G. salaris* from Ukraine is considered valid. No specimens of *G. salaris* collected from Ukrainian waters, however, have been confirmed by molecular methods.

#### 1978–Norway

The first confirmed observation of *G. salaris* in Norway was in 1975 at Sunndalsøra hatchery in Sunndalsøra, Møre and Romsdal County (today Forskningsstasjon for bærekraftig akvakultur, Sunndalsøra) ([63], and see [64–66]). This first identification was confirmed by Malmberg [67]. In the same year (1975), *G. salaris* was found in the Rivers Lakselva and Ranaelva following the high mortality of Atlantic salmon parr in these rivers [68]. Studies suggested that this parasite had been introduced, most likely from Sweden on several occasions [69–72] and that the Atlantic strain of *S. salar* was more susceptible to infection than the Baltic strain [5]. Four anthropogenic introduction routes have been suggested based on livestock movement records [64, 65]. Later studies [13] analysing sequences of COI, recovered three different haplotypes in Norway that were congruent with three of the suggested historical introduction routes (see [13] for details).

From 1975 to 2019, pathogenic strains of *G.* *salaris* were detected on Atlantic salmon fingerlings/parr in 51 rivers, 13 hatcheries/farms with Atlantic salmon parr/smolts and 26 hatcheries/farms with rainbow trout  [73, 74]. The last detections occurred as late as 2015 when the parasite was detected in the Kitdalselva River, in Troms County, during a rotenone treatment and very recently in 2019 when the Selvikvassdraget River (Vestfold and Telemark county) in the Drammen infection region was found infected [73, 74]. Arctic charr has also been found to carry *G. salaris* in Norway, and both strains that are pathogenic and non-pathogenic to Atlantic salmon have been detected on this host [75, 76].

In 1983, gyrodactylosis was declared a notifiable disease in Norway, and the policy of the Norwegian Authorities is to eradicate *G.* *salaris* from infected watersheds and farms  [77]. In farms, the parasite is eradicated by eliminating the hosts, while in rivers, the most common eradication measure has been the use of rotenone. In addition, the use of acidified aluminum sulphate, which kills the parasite without killing the host [78, 79], was used to treat the Lærdalselva River, Sogn og Fjordane County, and this river was declared free of infection with *G. salaris* in 2017. Low concentrations of sodium hypochlorite have also been shown to eliminate *G. salaris* in infection trials [80] and is now being tested as a treatment method for *G. salaris*. As of today, *G.* *salaris* has been confirmed eradicated from 39 rivers and from all hatcheries/fish farms where it has been present [73, 74]. In an additional four rivers, eradication measures have been completed, but eradication has not yet been confirmed (a river is declared free of infection about five years after treatment, the number of years depending on ages of smoltification in a particular river). At the end of 2019, the parasite was confirmed present in eight Norwegian rivers [73, 74].

In conclusion, the identification of *G. salaris* from Norway has been confirmed by many authors, using both morphological and molecular methods (e.g. [13, 24, 67]). Numerous reference sequences, especially COI sequences, are deposited in GenBank. As mentioned above, Hansen et al. [13] characterised three different mitochondrial haplotypes of *G. salaris* from Atlantic salmon in Norway. All infections detected in the Norwegian surveillance program for *G. salaris* [73, 74] have been confirmed by morphological and, subsequently, by molecular methods, and in the last 10 years or so by a combination of these methods.

#### 1983–Russia (including the Republic of Karelia but not Kaliningrad)

Specimens of *Gyrodactylus* sp. were collected by Ergens and Rumyantsev (unpublished data) in June 1972 from *S. salar* caught in Lake Ladoga, Republic of Karelia [39]. A subsequent re-examination of these specimens and a comparison with the re-described type material of *G. salaris* confirmed that the Karelian material was *G. salaris* (see [39]). Although the first record of *G. salaris* in Russia appears to have been made by Yekimova [81] working in the Pechora River, a subsequent re-examination of the specimens suggested that this was a misidentification [82, 83]. One year after the re-description of *G. salaris*, its occurrence on *S. salar* from the Pyalma River, Lake Onega, Republic of Karelia was reported [84]. *Gyrodactylus salaris* has also been recorded on salmon from the Keret River with prevalences approaching 100% and with mean intensities of approximately 300 parasites fish^−1^, suggesting that this parasite may be a factor in the decline of the salmon parr population in this river [82]. The population of *G. salaris* in the Keret River in Russia was suggested to have originated from the White Sea and to have spread by anthropogenic activities following an epidemic in the White Sea salmon stock [5, 30, 64, 69]. The date of this transfer was unknown until a mtDNA-based analysis was conducted by Kuusela et al. [86]. Following mitochondrial characterisation, the population of *G. salaris* in the Keret River was said to have originated from the Vyg (White Sea) hatchery between 1986 and 1989, when native salmon juveniles were transported by helicopter [86]. Kuusela et al. [86] and Ieshko et al. [87] suggested that the same canvas bag had been used to transfer fish to Lake Onega, where the parasite normally resides and does not cause any damage. The presence of *G. salaris* has also been recorded from the landlocked salmon population in the Pistojoki River, Lake Kuitozero [16], but this strain of *G. salaris* is most likely to have originated from rainbow trout that were stocked into fish farms in Kuusamo, Finland, upstream of the Pistojoki River [86]. The molecular identification of *G. salaris* from Russian *S. salar* has been confirmed by a number of authors [16, 24, 30, 64, 88].

Recently, *G. salaris* was also diagnosed from the Tuloma River, near Murmansk, Murmansky oblast, by the OIE reference laboratory [89]. The parasite was found on both wild salmon in the river and its tributaries, but also on rainbow trout in farms located in the river. 

Given the size of the Russian landmass, future studies might consider dividing the country into zones when mapping the occurrence of *G. salaris*. Defining these “zones” is not a simple matter and may be restricted to *G. salaris*-positive watersheds, as there are no geographic features that would otherwise limit its spread across the entire country. *Gyrodactylus salaris* has not been reported from the Russian exclave Kaliningrad, which is positioned between Poland and Lithuania.

In conclusion, the identity of *G. salaris* on *S. salar* in Russia has been confirmed by morphology and by multiple molecular-based studies and the record is considered valid.

#### 1983–Georgia

Malmberg [31] suggested that *G. salaris* was also present in Georgia, given that the description of *Gyrodactylus* sp.* sensu* Ergens, 1983 was shown to be a synonym of *G. salaris*. The report of this species from *S. trutta fario* collected from the Chernorechenskoye fish farm in 1978 by Ergens [39], therefore, is considered as valid, although future collections should, additionally, be verified by molecular-based approaches.

In conclusion, based on Malmberg’s [31] re-evaluation of Ergens [39] material and the drawings presented in Ergens [39] publication, which appear to be consistent with the morphology of *G. salaris*, the record is considered valid.

#### 1987–Finland

Although Rintamäki [90] reported the presence of *G. salaris* on Baltic salmon dating back to 1984, the first official record of this parasite from Finnish fish farms was published in 1987 by Rimaila-Pärnänen and Wiklund [91], who reported an infection on 18 fish farms that were studied between 1986 and 1987. Rintamäki [90] reported moderate to heavy infections of *G. salaris* on Baltic salmon from the Ossauskoski fish farm situated on the Kemijoki River, resulting in 8% mortality in the 1-year-old fish stocks. The occurrence of *G. salaris* from salmon fish farms connected to the Iijoki and Kemijoki rivers, and also reported for the first time from Finland, on rainbow trout, presented no clinical signs of disease [92]. Koski and Malmberg [47] carried out an additional investigation on *Gyrodactylus* specimens collected from rainbow trout and salmon (13 out of 33 farms were *Gyrodactylus* positive; 2416 rainbow trout and 1019 rainbow trout examined) in northern Finland, and the results confirmed the finding of *G. salaris* on salmon and rainbow trout without linked mortality. Identification was confirmed by Malmberg on the basis of morphology of ammonium picrate glycerine-prepared specimens. During these surveys, these researchers also found *Gyrodactylus lavareti* Malmberg, 1957 only on rainbow trout in a mixed infection with *G. salaris* (see [47]). The presence of *G. salaris* originating from Finland has also been confirmed several times by molecular analysis (see [16, 23, 88]). During the course of the present study, ten additional *Gyrodactylus* specimens from *O. mykiss* reared in the Jyväskylä region donated by Prof. E. Tellervo Valtonen were confirmed as *G. salaris* by both morphology and molecular analysis (the results are also presented in Table 1 in [21]).

In conclusion, the presence of *G. salaris* has been verified through multiple morphology ([47]; personal observation) and molecular-based studies [16, 23, 88]. Parts of Finland, however, have been declared *G. salaris*-free under European Commission (EC) decision (see Fig. 1 and later section on *G. salaris*-free states [92]). Fin samples from wild fish and farmed stock within the *G. salaris*-free and buffer zones are examined yearly [93, 94].

#### 1990–Germany 

Lux [95] was the first to report *G. salaris* in Germany from a survey of rainbow trout farms in the Brandenburg, Saxony and Thuringia districts. Although drawings consistent with *G. salaris* are presented in the account, Bakke et al. [2] were doubtful, suggesting that a molecular-based study was urgently needed to confirm their identity. In 2003, however, Cunningham et al. [23] acquired a single specimen from a rainbow trout farm in Berlin and confirmed it as being *G. salaris* by analysis of the ribosomal ITS and IGS region. In 2005, Dzika et al. [96] sampled a rainbow trout pond at Rogg in Bavaria, on a tributary of the Danube River, and reported finding *G. salaris* alongside *G. derjavinoides* Malmberg, Collins, Cunningham et Jalali, 2007, *G. truttae* Gläser, 1974 and *G. teuchis*. The accuracy of the *G. salaris* drawings, notably those of the marginal hooks, questions the validity of the identification of the specimens in this particular study, given that no reference specimens were deposited in a national collection, nor was molecular analysis conducted. During the present study, 20 specimens of *Gyrodactylus* from *O. mykiss* reared in Germany were kindly donated by Professor. Ewa Dzika. These specimens were mounted in ammonium picrate glycerine and confirmed as *G. salaris* by morphological identification only, supporting the findings of Dzika et al. [96], at least for *G. salaris* (personal observation).

In conclusion, the *G. salaris* status of Germany is considered to be valid and is based on the morphology of hooks presented in the original report of Lux [95], the molecular result of a single specimen made by Cunningham et al. [23] and the personal examination of a further 20 ammonium picrate glycerine specimens donated by Prof. Dzika from farmed *O. mykiss* in Germany.

#### 1997–Denmark

Malmberg [60] conducted a *Gyrodactylus* survey in three Danish rainbow trout hatcheries and reported the presence of two unidentified *Gyrodactylus* species that were very different to the highly pathogenic *G. salaris*. Although these most likely represented *G. derjavinoides* and *G. truttae*, both species were still undescribed at the time. In 1997, Buchmann and Bresciani [97] published the first official report of *G. salaris* on Danish rainbow trout, which was found to co-occur alongside *G. derjavinoides*. A small number of specimens were recovered from farmed fish at four farms and were identified based on their morphology from ammonium picrate glycerine-prepared material. Later, Nielsen and Buchmann [98] confirmed the presence of *G. salaris*, alongside *G. derjavinoides*, from eight rainbow trout farms during an 11-month sampling, using both morphology (*n* = 190 specimens from rainbow trout farmed in 5 counties; no drawings, however were presented) and molecular-based approaches (*n* = 26 confirmed by ITS restriction fragment length polymorphism [RFLP]:* Hae*III restriction of the ITS region of the rDNA gene) and by host. Although the latter study found only *G. salaris* and *G. derjavinoides*, an earlier study on Danish brown trout and other salmonids found two other species, i.e.* G. truttae* and *G. teuchis* (see [48, 99]). Lindenstrøm et al. [100] established a culture of *G. salaris* from farmed rainbow trout on the Vejle Å River, and this particular variant (*Gx*) was experimentally shown to exhibit low virulence towards Atlantic salmon. During a survey of wild Atlantic salmon from the Fladså River (Ribe Å River system), only one specimen of *G. salaris* was found, which was identified by morphological and molecular analyses [101]. Bakke et al. [2] suggested that there are no *G. salaris* epidemics on Danish wild salmon, probably because the variants of *G. salaris* from rainbow trout present in Denmark do not reproduce on Danish salmon populations, or due to the scarcity of wild salmon in Danish watersheds. There are only four Atlantic salmon rivers in Denmark, i.e. the Gudenå, Haderup, Skjern and Varde rivers (www.salmonatlas.com).

In conclusion, the combined morphology and molecular-based studies of Nielsen and Buchmann [98] and Jørgensen et al. [101], which found multiple specimens of *G. salaris* at farms in at least five counties and at different points in time, supports the *G. salaris*-positive status of the country.

#### 2003–Latvia

Specimens of *Gyrodactylus* (*n* = 2) collected from Baltic salmon from a fish farm near to the River Gauja were identified as *G. salaris* by sequencing of COI and found to carry a unique mitochondrial haplotype (haplotype D) by Hansen et al. [13]. This haplotype clusters with haplotypes A and B (from Norway and Sweden) and haplotype C (Sweden only), forming a single clade of *G. salaris* strains that only infects Atlantic salmon [13]. Later, Hansen et al. [26] added further information by analysing the IGS from the same Latvian specimens, finding the same IGS arrangements that were typical for *G. salaris* from Norway.

In conclusion, the *G. salaris*-positive status of the country is considered valid and is based on the COI sequencing of two specimens of *G. salaris* collected from farmed Baltic salmon. No morphological assessment of the specimens was provided in the study by Hansen et al. [13].

#### 2007–Poland

The first survey of *Gyrodactylus* on Polish salmonids was made from a fish farm and from the Soła and Czarna rivers by Prost [102], who found two species: *G. derjavinoides* from *S. trutta fario*, *O. mykiss* and *S. fontinalis*; and *G. truttae* from *S. trutta fario.* Subsequently, Rokicka et al. [103] reported finding specimens representing three molecular forms belonging to the *G. salaris*/*G. thymalli* group that were collected from Polish rainbow trout, sea trout (*S. trutta trutta* L.) and grayling from tributaries of the Vistula River, near Pomerania province. Identification of the forms was based on a PCR–RFLP analysis of the nuclear ITS fragment of rDNA. These three forms were represented by: (i) an ITS type which was only found on grayling; (ii) a heterogenic *G. salaris* type previously described by Lindenstrøm et al. [100] found on rainbow trout and sea trout; and (iii) a form found on rainbow trout, which was a complementary homozygous clone differing by three nucleotides [104]. The molecular identification was supported by a parallel morphometric analysis and from the drawings presented in Rokicka et al. [103].

In conclusion, the molecular identification and morphological analyses of multiple specimens of *G. salaris* collected from rainbow trout farms throughout Poland supports the *G. salaris*-positive status of the country.

#### 2007–North Macedonia (Macedonia until February 2019)

*Gyrodactylus salaris* has been confirmed present on Ohrid trout, *Salmo letnica* (Karaman), and from rainbow trout, both collected from a fish farm located on the Vardar River in the Aegean Sea basin, North Macedonia [88, 105]. The specimens were identified as *G. salaris* by sequencing of the ITS. While containing almost identical ITS sequences (differing by only 4 mutations), the COI sequences from specimens from the two hosts were highly divergent. The COI sequence of the specimens from the Ohrid trout was closely related to the haplotypes common on rainbow trout (e.g. AF479750), while the sequences from the rainbow trout were shown to be highly divergent (0.187 ± 0.012 Kimura 2-parameter distance) and said to be the result of introgression of mitochondria from another species into the genome of *G. salaris* [105].

In conclusion, North Macedonia is confirmed as *G. salaris*-positive based on the sequencing-based analyses of Kuusela et al. [88] and Ziętara et al. [105]. No morphological analyses were conducted.

#### 2009–Italy

A survey of five rainbow trout farms from four different regions in central and northern Italy by Paladini et al. [32] found that fish were infected with four species of *Gyrodactylus*, including *G. salaris* and three other (*G. derjavinoides*, *G. teuchis* and *G. truttae*). The specimens were collected throughout 2004–2005, and the morphological identification was confirmed by molecular analysis (sequencing of ITS2 and partial COI) (part of the molecular results are also presented in Table 1 in Shinn et al. [21]). An additional archived sample of formalin-fixed rainbow trout mucus scraped from infected fish dating back to 2000 was also found to contain *G. salaris.* Although these latter specimens were identified by morphology only, this confirmed that *G. salaris* had been in the country since at least 2000 and had persisted without causing any ascribed mortality [32]. For the current study, between the period 2008 and 2009, 27 samples of *Gyrodactylus* were collected from 20 Italian rainbow trout farms located in seven different regions (Friuli-Venezia Giulia, Lombardy, Piedmont, Trentino-Alto Adige, Tuscany, Umbria and Veneto) throughout the central and northern regions of Italy. Of these, 22 of the 27 (81.5%) samples collected were positive for the presence of *Gyrodactylus* spp. at low intensities of infection (4–30 parasites fish^−1^). *Gyrodactylus salaris* and *G. derjavinoides* were found in 17 samples from all seven regions; only two specimens of *G. truttae* were found, one in a sample from Veneto and one in a sample from Trentino-Alto Adige. *Gyrodactylus teuchis* was the predominant species found in all 22 *Gyrodactylus*-positive samples from all seven regions [21, 106]. The origin of *G. salaris* haplotype F in Italy [32] may be attributed to the trade in rainbow trout, given that this haplotype is common on rainbow trout in several European countries, including Denmark (see, e.g. [16, 104]).

In conclusion, the study of Paladini et al. [32] conducted on material collected from five rainbow trout farms across Trentino Alto Adige, Tuscany, Umbria and Veneto confirmed the identity of *G. salaris* based on 35 specimens prepared for morphology, eight specimens sequenced for ITS and seven specimens sequenced for mtCOI.

#### 2010–Estonia

A survey on triploid Atlantic salmon from the Baltic basin showed a high susceptibility to *G. salaris* infection [107]. This fish population and associated parasites originated from a hatchery in northern Estonia, situated on the Kunda River, Gulf of Finland, Baltic Sea [107]. Identification of *G. salaris* was confirmed by molecular analyses, including sequencing of the ITS rDNA and mtCOI [107]. Although there is no doubt regarding the identification of these specimens, no morphological data are available. According to the mtDNA analyses reported in Ozerov et al. [107], the closest relatives to the Estonian strain of *G. salaris* is the strain of *G. salaris* found in Genevadsån on the Swedish west coast and those collected from the Raasakka hatchery, Iijoki, Gulf of Bothnia, Finland.

In conclusion, the *G. salaris*-positive status of the country is supported by the molecular study of Ozerov et al. [107].

#### 2016–Romania

The first report of *G. salaris* from Romania is from the study of Hansen et al. [15], who reports their findings from a survey of *Gyrodactylus* spp. on salmonids (rainbow, brook and brown trout) in Romanian fish farms and from one river. Of the 187 specimens recovered from the fish, a sub-sample of 76 specimens were identified through sequencing of the ITS2. Of these, 31 were identified as *G. salaris*, and *G. salaris* was found on all three hosts examined. Apart from in experimental infections, this is the first report of *G. salaris* being found on brook trout. Morphological analyses were performed on a sub-sample of these to complement the molecular analyses. MtCOI sequences were also obtained from all specimens identified as *G. salaris*, and four haplotypes were recovered. As all these haplotypes were new to science it was impossible to establish the origin of infection of *G. salaris* in these fish farms. It was, however, speculated that they might have been introduced via the import of rainbow trout.

In conclusion, Romania is considered to be a *G. salaris*-positive state based on the molecular and morphological study conducted by Hansen et al. [15].

### Confirmed *Gyrodactylus salaris*-free states based on surveillance

#### UK (including Northern Ireland)

Following the catastrophic events resulting from the introduction of *G. salaris* to Norway, *G. salaris* was made a notifiable pathogen in the UK (including Northern Ireland) in 1987 under the Diseases of Fish Acts 1937 and 1983, which can impose movement restrictions on fish stocks from fish farms, rivers or entire catchments [1]. This act does not extend to Northern Ireland. Following notification, a survey of seven rivers and 17 fish farms in Northern Ireland [108] and a parallel investigation of 63 fish farms and 164 wild salmonid sites throughout the UK by Shinn et al. [109] were initiated to establish the *G. salaris* status of each. Neither survey found *G. salaris* or the morphologically similar *G. teuchis*, but the surveys did find *Gyrodactylus arcuatus* Bychowsky, 1933 and *G. caledoniensis* Shinn, Sommerville et Gibson, 1995 from *S. salar*; *G. derjavinoides* from *O. mykiss*, *S. alpinus alpinus* (L.), *S. salar* and *S. trutta fario*; *G. truttae* on *S. trutta fario*; and a number of unidentified *Gyrodactylus* morphotypes from *S. alpinus alpinus* and *S. salar*. Mandatory surveillance programmes by the relevant fish inspectorate authorities within each constituent country continue to screen fish samples for *G. salaris* and other pathogens of concern. National contingency planning in the event of a *G. salaris* introduction began in 2006 in Scotland (www.scotland.gov.uk), in 2008 in England (www.oie.int) and Wales (http://wales.gov.uk) and in 2009 in Northern Ireland (www.dardni.gov.uk). The UK is officially a *G. salaris*-free zone under EC Decision 2004/453/EC and its subsequent amendments provided under EC Decision 2006/272/EC.

Although GB is *G. salaris*-free, there is a single report of *G. salaris* from *S. trutta fario* from Loch Leven, Scotland [110]. Malmberg [10] considered this to be a misidentification with *G*. *derjavinoides* or *G*. *truttae*, species that were both still undescribed at the time of publication. Salmonids from Loch Lomond were sampled during the study of Shinn et al. [108], but no specimens of *G. salaris* were found. Specimens of *G. thymalli* collected from *T. thymallus* from the Test River, were confirmed by molecular methods as *G. thymalli* [21, 27].

There are ongoing programmes of *G. salaris* health surveillance conducted by the fish health inspectorates (FHI) within each of the four countries of the UK (England: FHI, Centre for Environment, Fisheries and Aquaculture Science (Cefas), www.cefas.co.uk and www.gov.uk; Scotland: Marine Scotland, www.gov.scot; Wales: Natural Resources Wales, www.naturalresourceswales.gov.uk; and Northern Ireland: Department of Agriculture, Environment & Rural Affairs, http://www.daera-ni.gov.uk).

In conclusion, the UK (including Northern Ireland) and the territories of Guernsey, Jersey and the Isle of Man are considered to be *G. salaris*-free.

#### Finland—selected water catchments

Parts of the Finnish territory have been declared *G. salaris*-free under EC Decision 2010/221/EU [94]. These regions include the water catchment areas of the Tenojoki and Näätämönjoki, whilst the Paatsjoki, Luttojoki and Uutuanjoki water catchment areas are considered to be buffer zones (see Fig. 1).

In conclusion, although Finland is a *G. salaris*-positive state, parts of the country are recognised to be *G. salaris*-free; samples from wild fish and farmed fish from within the *G. salaris*-free and buffer zones are examined yearly [93].

#### Republic of Ireland

The Republic of Ireland is declared *G. salaris*-free under the EC Decision 2004/453/EC based on evidence that its government submitted to the EC. National surveillance for *G. salaris* is conducted by the Marine Institute (www.fishhealth.ie).

In conclusion, *Gyrodactylus salaris* is not known from the country.

### States where the *G. salaris* status requires confirmation

#### 1961–Slovakia

Ergens [37, 38] recorded the presence of *G. salaris* in Slovakia (formerly Czechoslovakia) from brown trout from the Topl’a River, near the town of Bardějov in the north-east of the country. A later re-examination of this material [39] found that the species in question was *G. truttae*, a species not described at the time of Ergens’ original study. The identity of *G. truttae* was evident from the measurements of the haptoral hard parts [39, 111]. The record of *G. salaris* from Slovakia, therefore, is not considered valid.

In conclusion, based on the information to date, the record of *G. salaris* is not valid.

#### 1967–Bosnia and Herzegovina

The first two reports of *G. salaris* from *S. salar* cultured in Bosnia and Herzegovina date back to 1967 [112, 113]. Žitňan and Čanković [114] later recorded *G. salaris* from rainbow trout and brown trout from the Buna and Pliva rivers, which run through two fish farms sited at Blagaj and Jezero, near the towns of Jajce and Mostar, respectively, and from Adriatic trout *Salmo obtusirostris* Heckel, from a site on the Buna River. Ergens [39] listed the species of *Gyrodactylus* collected from the Buna and Pliva rivers as *Gyrodactylus truttae* but did not emphatically state which hosts the samples were collected from. Ergens [39], however, lists brook, brown and rainbow trout among the salmonid hosts examined in his Eurasian review of *Gyrodactylus* species infecting salmonids. Also, it is not clear whether Ergens [39] based his identities on a re-examination of the specimens collected during the earlier study or on the assessment of material that was subsequently acquired. Although Ergens [39] comments on *G. salaris* in this paper, he does not comment on its occurrence in Bosnia and Herzegovina, leading to the conclusion that he regarded the species to be absent at the time. The validity of the *G. salaris* records were also questioned by Bakke et al. [115], an opinion based on Tanum’s [66] assessment of the material, who considered the reports of *G. salaris* from *O. mykiss* and from *S. trutta fario* as misidentifications. This did not, however, apply to the record of *G. salaris* from *S. obtusirostris.* Ergens [39] makes no mention of this latter record or host in his study, suggesting that either the specimens were not available for re-examination or the record was overlooked. Following the study of Žitňan and Čanković [114], two further reports of *G. salaris* infections from the skin and fins of rainbow trout fry were recorded from three fish farms situated at Blagaj near the town Jajce, Ljuta (near Konjic) and Jezero (near Mostar), where mortalities of 3–5% were reported, and also from the Ribnik River [116, 117]. Although Imamović [117] reported the presence of *G. salaris* in Bosnia and Herzegovina, it is not possible to verify the true parasite species identity based on the drawings of the attachment hooks that are presented in the article.

In conclusion, based on the reports to date, the identity and validity of *G. salaris* in Bosnia-Herzegovina is questionable. No specimens of *G. salaris* collected from Bosnia and Herzegovina have been confirmed by molecular methods.

#### 1974–Czech Republic

The first record of *G. salaris* from Czechoslavakia was in 1958—a misidentification of *G. truttae* (see [37, 39]). Later in 1974, Tesarcik and Ivasik [61] reported the finding of *G. salaris* from a study which included the collection of brown trout from the north-Moravian Moravice River in the Czech Republic (= Czechia). In the absence of drawings included within their report, this record of *G. salaris* cannot be substantiated.

**Fig. 2:**
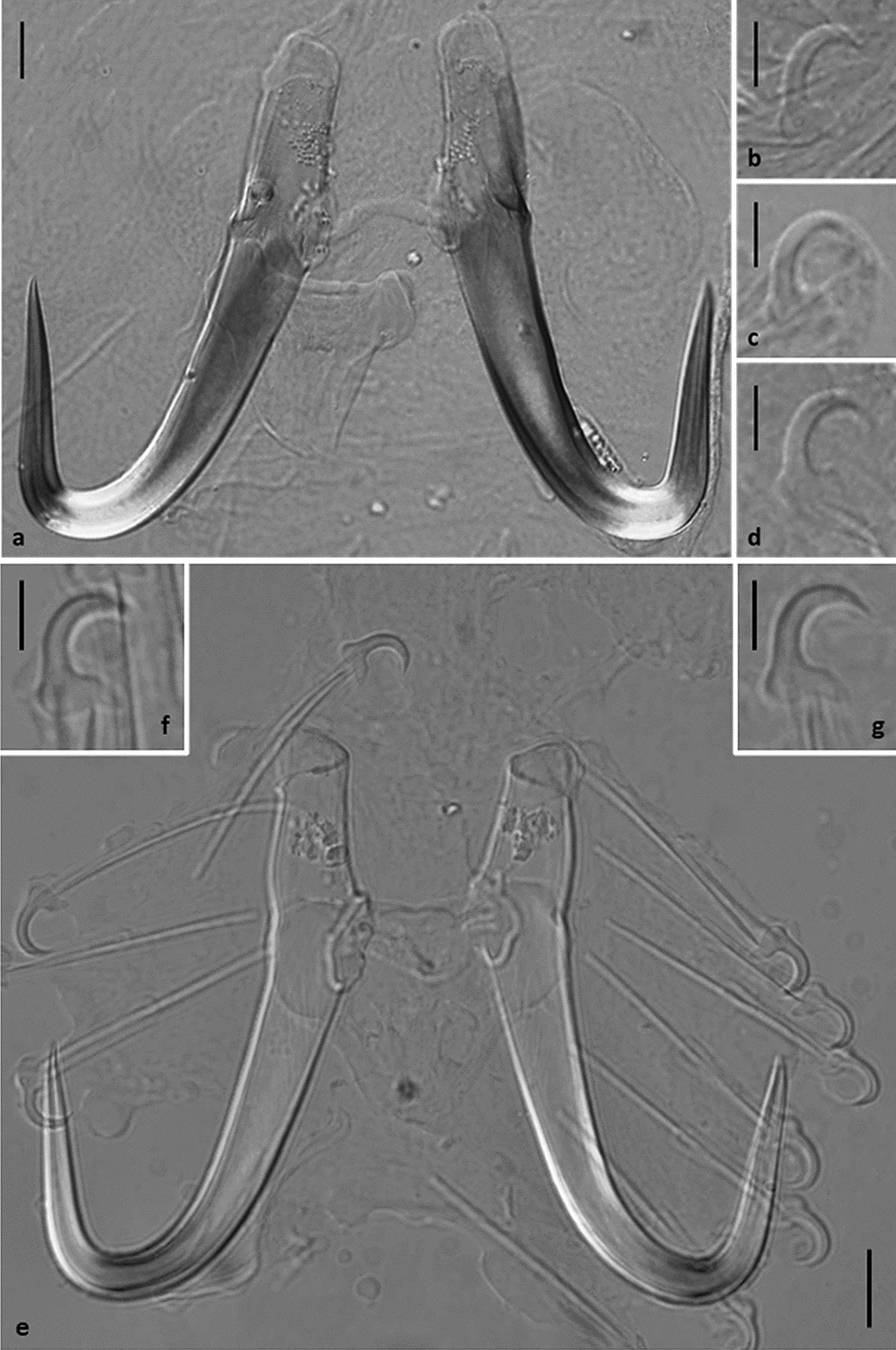
Light micrographs of a paratype specimen (acc. no. M-342, Institute of Parasitology, Czech Academy of Sciences) of *Gyrodactylus bohemicus* Ergens, 1992 from farmed *Oncorhynchus mykiss* (Walbaum) from the Czech Republic. **a** Hamulus complex, **b**–**d** marginal hook sickles. Scale bars: 10 μm (**a**) 5 μm (**b–d**). (Images were kindly provided by Dr. R. Kuchta). **e–g** Light micrographs of *Gyrodactylus salaris* Malmberg, 1957 from *Salmo salar* L. from Norway: **e** hamulus complex, **f**,** g** marginal hook sickles. Scale bars: 10 μm (**e**), 5 μm (**f**, **g**)

In the absence of molecular data, the discovery of a species morphologically similar to *G. salaris*, namely *Gyrodactylus bohemicus* Ergens, 1992 from farmed *O. mykiss* and *S. fontinalis* in the Czech Republic [118], raised the question of whether this represented a discrete species or a misidentification of *G. salaris*. Ergens [118] commented on the morphological similarities of *G. bohemicus* with *G. thymalli* and *Gyrodactylus magnus* Konovalov, 1967, but made no reference to *G. salaris*. Bakke et al. [2] commented on the morphological similarity of *G. bohemicus* to *G. thymalli* and to the variant of *G. salaris* on rainbow trout. This was also discussed by Lindenstrøm et al. [100] in their assessment of *Gx*, a *G. salaris* variant, to other morphologically similar species. Ergens [39] in his review of *Gyrodactylus* species parasitising salmonids and thymallids in Eurasia made no reference to the occurrence of *G. salaris* in the Czech Republic at the time nor to the study of Tesarcik and Ivasik [61]. Although three paratypes of *G. bohemicus* are deposited in the monogenean collection maintained by the Institute of Parasitology, Czech Academy of Sciences (acc. no. M-342), these valuable specimens were not available for scientific loan and direct first-hand examination. Pictures of the paratypes of *G. bohemicus* were, however, taken and provided by Dr Roman Kuchta for the current study, and morphological examination of their attachment hooks suggests a very close similarity to those of *G. salaris* (current study; see Fig. 2). Further comments on this, however, must wait until more specimens can be collected and evaluated through a molecular comparison with congeners. It is for these latter reasons that Bakke et al. [2] cautiously suggested that *G. salaris* is probably absent from the Czech Republic, but comments that a detailed study to establish its presence or otherwise would be worthwhile. In a study carried out by Matejusová et al. [34], a single specimen of *Gyrodactylus* was recovered from a brown trout sampled from the Vlára River. The identity of this specimen, however, was not clearly defined and it was referred to as *G. salaris*/*G. thymalli* (see [34]), since it was not possible to reach any other conclusion. As grayling, but not salmon, is present in the Vlára River [119], the specimen sequenced is considered to be *G. thymalli* originating from grayling. Although accidental infections of *Gyrodactylus* do occur, and the cross transfer of species between cohabited hosts even during short periods of holding has been demonstrated [120], *G. thymalli* has not been previously reported from brown trout. The name *G. thymalli*, however, has never been used for parasites on other hosts than grayling, so by definition, the specimen collected and analysed by Matejusová et al. [34] would be regarded as *G. salaris*. If, however, the same criteria detailed by Hansen et al. [15] for identifying *Gyrodactylus* species are applied, then in cases like this, where the morphology and ITS sequences from a specimen corresponds to *G. salaris*/*G. thymalli*, and where the COI sequences cannot be assigned to a previously known haplotype associated with a specific host species, then identification is implicitly host-based. The name *G. thymalli* is thus so far used for parasites from *T. thymallus* only, while specimens from other hosts are named *G. salaris*; i.e. *G. salaris* is defined from salmon or from rainbow trout. Although the COI was not sequenced in this particular instance, the ITS sequence and morphology corresponded to *G. salaris*, and the specimen was collected from brown trout and not grayling, hence by definition it should be classified as “*G. salaris*”.

There are, however, other reports of “*G. salaris*” from the Czech Republic, which may represent misidentifications of either *Gyrodactylus derjavinoides* Malmberg, Collins, Cunningham et Jalali, 2007 and/or *G. truttae*, neither of which had been discovered and described at the time the relevant “*G. salaris*” report was made. These include the record of *Gyrodactylus* specimens from brown trout from the Osoblaha River [121] and from rainbow trout from a fish farm near the town of Český Krumlov [122]. In a study by Řehulka [123], specimens of brown, rainbow and brook trout were infected with *G. salaris sensu* Ergens, 1961, which later was determined to be a misidentification of *G. truttae*, whose attachment hooks vary markedly in size from those of *G. salaris* (see [118, 124]).

In conclusion, the host-based argument of the specimen identified by Matejusová et al. [33] as being *G. salaris*/*G. thymalli* from *S. trutta* leaves room for doubt as grayling is found within the country; as such the presence of *G. salaris* in the Czech Republic cannot be confirmed.

#### 1991–Spain

Two pharmaceutical trials conducted in Spain on the species of *Gyrodactylus* collected from rainbow trout from Carballo, La Coruña, were identified, on the basis of hook morphology, by Professor Göran Malmberg (University of Stockholm) as *G. salaris* (see [125, 126]); this was, however, at a time before the existence of *G. teuchis* was known. The authors of this earlier work were contacted, but the slides from this study no longer exist. As with the reports for France and Portugal, it is likely that these specimens were *G. teuchis* and were mistaken for *G. salaris*. A sample of 60 *Gyrodactylus* specimens collected for the current study from rainbow trout fingerlings from a farm in the Galicia region of Spain were all identified as *G. teuchis* by morphology only; the manner in which the specimens were fixed on the farm did not, unfortunately, permit their analysis by molecular methods.

In conclusion, in the absence of molecular data the identity made by Professor Malmberg cannot be substantiated, and as such the *G. salaris* status of the country requires confirmation.

#### 1996–France

The first record of *G. salaris* in France (and also in Portugal) was made by Johnston et al. [127] with reference to material collected from rainbow trout and identified using morphology and a DNA probe based on the V4 region of the ribosomal small subunit. The subsequent discovery of *G. teuchis*, a species which has morphological similarities with *G. salaris*, makes the validity of this earlier *G. salaris* finding questionable [40]. This latter study and that of Cunningham et al. [41]—which looked at material collected from a large scale survey of Atlantic salmon, rainbow trout and brown trout farms‒did not find *G. salaris*, and therefore was unable to support the suggestion that France is a *G. salaris*-positive state. The report of *G. salaris* from France was, therefore, most likely the result of a misidentification between *G. salaris* and *G. teuchis*.

In conclusion, the *G. salaris*-positive status of France is questionable and requires validation from the molecular analysis of further *Gyrodactylus* specimens.

#### 1996–Portugal

The assessment of Johnston et al. [127] of the *Gyrodactylus* specimens collected from farmed Portuguese rainbow trout was based on both morphological and molecular data. The specimens, however, were initially fixed in buffered formalin and then rinsed in 70% ethanol before being assessed. It is likely that the formalin fixation would have prevented flat preparations of *Gyrodactylus* and, therefore, a clear view of the marginal hooks, which are considered the key morphological feature upon which to identify species. *Gyrodactylus teuchis* was an unknown species at the time of study and given the morphological similarities between this and *G. salaris*, it is possible that the subtle differences in hook shape were not recognised as deviating from those of *G. salaris*. Subsequent studies by Lautraite et al. [40] and Cunningham et al. [41] described *G. teuchis* and its discrimination from *G. salaris* by morphology and differences in PCR–RFLP patterns of the ITS1, 5.8S gene and ITS1 regions. A survey of salmonids throughout France by both latter studies led to the conclusion that France was most likely a *G. salaris*-free state and that the original report was a result of a misidentification. Although Eiras [128] conducted a survey on several Portuguese rainbow trout and brown trout farms, no specimens of *G. salaris* were found. Johnston et al.’s [127] identification of *G. salaris* from Portugal, therefore, remains in doubt until demonstrated otherwise.

In September 2007, three specimens of *Gyrodactylus* were recovered from a sample of 20 Portuguese rainbow trout. All three specimens were confirmed, during the current study, as *G. teuchis* by morphological and molecular examinations. The sequences were submitted to GenBank under acc. no. MN853657.

In conclusion, Portugal’s *G. salaris* status is questionable and requires validating through a survey of its *Gyrodactylus* fauna.

#### 2001–Kazakhstan or Tajikistan

The Natural History Museum (NHM), London, maintains a “host-parasite” database (www.nhm.ac.uk) which is populated with published parasite data up to and including 2002. On this database, there is a record of *G. salaris* from Aral trout, *Salmo trutta aralensis* Berg, from Kazakhstan linked to a paper by Gvozdev and Karabekova [129]. From this reference, however, Amu-Darya trout *Salmo trutta oxianus* Kessler is listed as a host for *G. salaris* from the Kafirnigan River, in Tajikistan, which could have been misidentified as *Gyrodactylus derjavini* Mikhailov, 1975, the only “other” *Gyrodactylus* species previously recorded from this host (see [39]; www.gyrodb.net; Prof. Margaritov N.M., personal communication). An earlier, similar reference by Gvozdev and Karabekova [130] does not mention *G. salaris* within the 43 listed species of *Gyrodactylus*, although the abstract indicates that 48 *Gyrodactylus* species are listed. The validity of the *G. salaris* report from Kazakhstan or Tajikistan is questionable, and although attempts have been made to contact the authors, no communication has been established. This report cannot be confirmed until further detailed information on this report is available, or specimens can be obtained and assessed.

In conclusion, the *G. salaris* status of Kazakhstan or Tajikistan is questionable and remains to be investigated.

### Comments on other European states

#### 1967–Moldova (including Transnistria)

*Gyrodactylus salaris* has not been reported from Moldova. It has, however, been reported from *S. trutta* from the Seret River, Ukraine [59], which is a tributary of the Dniester River, which forms the eastern boundary of Moldova and the breakaway territory of Transnistria. As this account did not include drawings, nor a link to specimens deposited in a national collection that can be re-examined, the record cannot be validated. On the NHM, London host-parasite database, *G. salaris* is reported from *S. trutta* in the “Ukraine, including Moldavia” with the report being accredited to Malmberg [31]. Moldova declared independence in 1991, with the constitution of Moldova being adopted in 1994; Malmberg [31], however, referred to this record as being taken from Ukraine.

In conclusion, although *G. salaris* specimens have been collected from brown trout from the Seret River on the border, in the absence of morphology and molecular-based data from specimens collected within national borders, the *G. salaris* status of the territory cannot be confirmed and must therefore await confirmation.

#### Austria

*Gyrodactylus salaris* has not been reported from Austria although a number of studies looking at the gyrodactylid fauna of various salmonids have been conducted ([131–133]; see Additional file 1: Table S1).

#### Bulgaria, Croatia, Lithuania and Turkey

Brief details of other *Gyrodactylus* species that have been found on salmonids in each country are provided in Additional file 1: Table S1.

## Discussion

The main host of *G. salaris*, the Atlantic salmon, has a wide distribution in Europe and can be found along the coasts of the North Atlantic, including the Baltic Sea and their range extends from the Bay of Biscay to the White Sea. Colonisation of northern Europe most likely occurred from the sea after the last glaciation event [134]. Although most species of *Gyrodactylus* are host specific or have a narrow host range [135], *G. salaris* seems to display lower host specificity and can colonise and reproduce on a range of salmonid hosts. It is assumed, however, that lower host specificity might be the result of the number of studies performed with this species compared to all other species of *Gyrodactylus*.

*Gyrodactylus salaris* has, under natural conditions in the wild, been recorded from *S. salar* (e.g. [39, 136]), *O. mykiss* [e.g. [137]], *S. trutta* (e.g. [44, 66]), *S. alpinus alpinus* (see [137–139]), *Salmo obtusirostris* (see [112]) and *Platichthys flesus* (see [139]), although the latter, as a non-salmonid species, has proven to be an unsuitable host [115]. The relative susceptibility of these hosts, and even of different populations of these hosts, to *G. salaris* varies, as does the pathology induced (e.g. [5, 28, 75, 76, 115, 140–144]).

Although *G. salaris* was initially classified as a List III pathogen under the European Council Directive 91/67/EEC regarding measures against certain diseases in aquaculture animals, it has since been removed following EC Directive 2006/88/EC, but remains on OIE lists as a “significant disease” and “notifiable pathogen” [1].

Of all the European countries, considerable stocks of wild salmon populations are present only in Norway, Scotland, Faroe Islands, Ireland and Iceland [145]. In other states within Europe, the number of salmon populations is small [146–148]. The dissemination of *G. salaris* across Europe and outside its native range appears mainly to be linked to movements of rainbow trout between countries [2, 7, 145]. This appears to be the case for most of the *G. salaris* reports from southern Europe, such as Italy and Romania, where salmon is not present, but *G. salaris* has still been recorded in many localities where rainbow trout is farmed [15, 32, 106]. There are 50 sovereign states within Europe, four of which are trancontinental, and six other states, and although most contain salmonid species, a number of smaller territories, such as Gibraltar, Malta, Monaco and Vatican City, do not. While the Republic of San Marino is considered to be salmonid-free by the on-line database FishBase (www.fishbase.org), Lake Faetano, a small artificial lake created in 1968 for recreational fishing, does contain rainbow trout and brown trout, and the *G. salaris* status of these stocks requires establishing. The lack of clinical signs of gyrodactylosis on species such as rainbow trout means that *G. salaris* infections may go undetected for many years, such as in Italy where *G. salaris* infections had persisted unknown for at least 9 years prior to its first official report [32]. This finding is an important consideration when moving salmonid stocks and calls for more rigorous biosecurity control measures in the trade and transfer of fish species from one country to another, or between different regions within one country. *Gyrodactylus cichlidarum* Paperna, 1968 on Nile tilapia, *Oreochromis niloticus niloticus* (L.), has been exported—undetected—with its host worldwide and has been responsible for the mass mortality of juvenile Nile tilapia reported from several countries outside its native origins in Africa [149].

*Gyrodactylus salaris* has been reported from 23 out of the approximately 50 recognised states throughout Europe (Table 1). Only 14 of these records, however, are considered valid, having been identified by either morphology, molecular studies or a combination of both methods, and only nine of these latter 14 reports have been confirmed by a combination of both molecular and morphological approaches (Table 1). The records of *G. salaris* from France, Portugal, Slovakia and Spain all appear to have been based on misidentifications, and although some additional specimens have been obtained from some of these countries, and found only to contain *G. teuchis*, larger numbers of samples are required before a definitive statement can be made. In the case of France, however, a robust survey conducted in Brittany and the Adour Basin was conducted [40], but only the morphologically similar species *G. teuchis* was found. Likewise, the report of *G. salaris* from Kazakhstan (or Tajikistan) is doubtful, and further samples are required for evaluation. The records of *G. salaris* from Bosnia-Herzegovina, Georgia, Moldova and Ukraine are all based on morphology only, and ideally these reports require confirmation by an appropriate molecular test. The only regions that are currently considered to be *G. salaris*-free are the Republic of Ireland, the UK (including Northern Ireland) and the Finnish water catchments of the Tenojoki and Näätämönjoki, where on-going government-based surveillance programmes continue to screen salmonids from key sites.

## Supplementary information

**Additional file 1: Table S1.** A list of additional European territories where *Gyrodactylus* have been found on salmonids.

## Data Availability

All data generated or analysed during this study are included in this published article.
