# Supplementary material for: Geographical distribution of Gyrodactylus salaris Malmberg, 1957 (Monogenea, Gyrodactylidae)
Source: Parasit Vectors. 2021 Jan 9;14:34. doi: 10.1186/s13071-020-04504-5 (PMC7796612; doi:10.1186/s13071-020-04504-5)
Supplement: Supplementary file 1 — Additional file 1: Table S1. A list of additional European territories where Gyrodactylus have been found on salmonids. [file 13071_2020_4504_MOESM1_ESM.docx]

**Table S1.** A list of additional European territories where *Gyrodactylus* have been found on salmonids.

| **European countries** | ***Gyrodactylus* species** | **Host** | **References** | **Status of**  ***G. salaris*** |
| --- | --- | --- | --- | --- |
| Austria (AT) | *G. teuchis* | *Stf* | [130-131] |  |
|  | *G. thymalli* | *Tt* | [131]^1^ | unknown |
|  | *G. truttae* | *Saa* | [132] |  |
| Bulgaria (BG) | *G. truttae* | *Om*, *Stf* | [152] | unknown |
| Croatia (HR) | *G. salmonis* | *Om* | [153] | unknown |
| Lithuania (LT) | *G. rarus* | *Om* | Host-parasite DB^2^ | unknown |
| Turkey (TR) | *Gyrodactylus* spp. | *Om* | [154] | unknown |

Note: *Om*: *Oncorhynchus mykiss*; *Saa*: *Salvelinus alpinus alpinus*; *Stf*: *Salmo trutta fario*; *Tt*: *Thymallus thymallus*. ^1^*G. thymalli* has been found on a grayling collected 1880 in Austria which was deposited in the fish collection of the Natural History Museum, Vienna, indicating that this parasite has been there for at least 130 years, long before its description in 1960 (Hahn C., *pers. comm.*); ^2^Host-parasite database of the Natural History Museum of London (UK): www.nhm.ac.uk.

**Supplementary references**

153. Kakacheva-Avramova D, Menkova I. Helminths in trouts (Fam. Salmonidae) from fresh-water basins. Vet Med Nauki. 1982;19:78–84. [In Bulgarian].

154. Zrnčić S, Oraić D. Legislation and health situation in Croatian aquaculture. Proceedings of the XV Convegno Nazionale S.I.P.I. Società Italiana di Patologia Ittica, 22^nd^-24^th^ October 2008, Erice (TP), Italy. 2008;68–9.

155. Ozkan Ozyer B. Legislation and health situation in aquaculture in Turkey. Proceedings of the XV Convegno Nazionale S.I.P.I. Società Italiana di Patologia Ittica, 22^nd^-24^th^ October 2008, Erice (TP), Italy. 2008;70–1.
